# HIV-related Stigma in Rural and Tribal Communities of Maharashtra, India

**DOI:** 10.3329/jhpn.v30i4.13291

**Published:** 2012-12

**Authors:** Carol Vlassoff, Mitchell G. Weiss, Shobha Rao, Firdaus Ali, Tracey Prentice

**Affiliations:** ^1^Department of Epidemiology and Community Medicine, University of Ottawa, 451 Smyth Road, Ottawa, Ontario, Canada;; ^2^Department of Epidemiology and Public Health, Swiss Tropical and Public Health Institute, University of Basel, Switzerland;; ^3^Department of Biometry and Nutrition, Agharkar Research Institute, Pune, India;; ^4^Alliance for South Asian AIDS Prevention, Toronto, Canada;; ^5^Institute for Population Health, University of Ottawa, Ottawa, Canada

**Keywords:** Community, HIV-related stigma, Rural health, Tribal, India

## Abstract

Stigma is a recognized barrier to early detection of HIV and causes great suffering for those affected. This paper examines HIV-related stigma in rural and tribal communities of Maharashtra, an area of relatively high HIV prevalence in India. The study used a mix of qualitative and quantitative methods to compare adult women and adolescents in a rural area, women in a rural area, and women in a tribal area. The respondents included 494 married women and 186 adolescents in a rural community and 49 married women in six tribal villages. HIV-related stigma was prevalent in all communities and was the highest among tribal and older respondents. High-risk behaviour was reported in both areas, accompanied with denial of personal risk. Our findings suggest that HIV may be spreading silently in these communities. To our knowledge, this is the first community-based study to make an in-depth assessment of HIV-related stigma in rural and tribal areas of India. By situating our findings within the broader discourse on stigma in the national and state-level data, this study helps explain the nature and persistence of stigma and how to address it more effectively among subcultural groups in India.

## INTRODUCTION

An estimated 2.5 million people are living with HIV/AIDS in India (1), and many more are thought to be living with undiagnosed infection due to high levels of stigma and discrimination (2). Fear of stigma hampers disclosure to others (3,4), and HIV-positive individuals are often blamed for their illness, causing a great deal of shame and suffering (5,6,7). Therefore, the reduction of stigma has been identified as a priority by India's National AIDS Control Organization (NACO).

India's National Family Health Survey-3 (NFHS-3), conducted in 2005-2006, provides the most comprehensive picture of India's HIV situation (1). HIV prevalence is higher in urban (0.35%) than in rural areas (0.25%) (1), and prevalence in rural areas appears to be rising (8,9). Even a relatively low prevalence in rural areas represents a large number of HIV-affected people, given that 72% of India's population of over 1.15 billion is rural. High rates of sexually transmitted infections (STIs) also contribute to HIV transmission (8,10).

The NFHS-3 reported limited knowledge about HIV: nationally, only 17% of women and 33% of men had a comprehensive knowledge about HIV prevention and transmission. Four issues focused on HIV-related stigma: (i) caring for an HIV positive relative; (ii) buying fresh vegetables from someone with HIV; (iii) allowing an HIV-positive female teacher, not visibly ill, to continue teaching; and (iv) willingness to reveal a family member's positive HIV status. Nationally, over half of the respondents gave non-stigmatizing replies to individual questions but only 34% of females and 37% of males did so to all four questions.

The HIV situation in India is complicated by several sociocultural factors, including social pressure on women to become pregnant soon after marriage, the belief that married women cannot contract HIV, and the cultural association of HIV with immoral sexual behaviour. Such norms make women extremely vulnerable, especially when husbands are HIV-positive (8,9). These factors also impede timely help-seeking among those at risk or already infected (7,9,11).

Apart from the NFHS-3 data, only a few studies have focused on HIV-related stigma in India, and most of these have concentrated on the perspectives of people with HIV (4,12,13). Community stigma was identified as a cause of discrimination toward people with HIV in a study in rural Pune district, Maharashtra but stigma was neither defined nor quantified (12). The study reported that people hid their HIV status until they were visibly ill, after which they were socially and physically isolated. HIV-positive women, including those infected by their husbands, were blamed more than men who received more social support and consumed most of the family's resources for care and treatment.

Although India's tribal populations are at considerable risk of STIs because of their relatively open sexual mores compared to traditional Indian society (14,15), limited attention has been paid to HIV among them. Previous studies have documented high rates of STIs, limited prevention and surveillance, low awareness, and widespread misconceptions about HIV. These factors, combined with inadequate healthcare, create a fragile situation susceptible to the rapid spread of HIV in most tribal areas (10,14,15). While it could be expected that HIV-related stigma would also be high, the NFHS-3 data did not show large variations between tribal and non-tribal respondents in this respect.

Given that stigma at the community level is widely acknowledged as a barrier to early HIV diagnosis and treatment, the need for a better understanding of its nature and extent, especially in India's rural and tribal areas, has been highlighted (7,12,16,17). The objective of this study is to explore this issue in rural Maharashtra, an area with high HIV prevalence, and to suggest how the response to HIV in rural and tribal communities could be enhanced. Although the NFHS-3 data included samples of individuals from different rural and tribal areas, no in-depth analysis of qualitative information has been published. To our knowledge, ours is the first community-based study to make an in-depth assessment of HIV-related stigma in rural and tribal areas of India.

## MATERIALS AND METHODS

### Study area

This study was conducted between October 2007 and March 2008 in Satara district where the antenatal clinic of the District Hospital in 2007 reported that about 2% of women were HIV-positive compared to an estimated 0.5% for Maharashtra as a whole (18). The same prevalence was reported for Satara district by NACO in 2006 (18). This relatively high prevalence has been linked to occupational risk factors for men, especially transport and trucking and temporary rural-urban migration (18).

The largest of the two study populations, the ‘rural’ community of 3,326 residents, was the site of a longitudinal study of women's reproductive health conducted between 1975 and 2008. HIV was included only in the 2007-2008 study. Undertaking community-level research on the sensitive issue of HIV-related stigma was facilitated by the researchers’ familiarity with and acceptance by the villagers.

The other study population belonged to six small tribal (*Adivasi*) villages located about 25 km from the rural community under study and were selected on the advice of three community development organizations. One of these organizations provided income-generating opportunities and HIV-related information to surrounding tribal communities.

### Research methods

This study employed a mix of qualitative and quantitative methods to explore perceptions of HIV and of those affected by it. In the rural area, qualitative data were obtained from three and two focus group discussions (FGDs) among male and female adults respectively. The FGD topics, posed by a moderator, concerned issues ranging from general HIV-related knowledge to more intimate subjects, such as knowing someone with HIV and sharing food. Quantitative data were collected by questionnaire interviews which also included open-ended questions for qualitative information.

In the tribal area, the same sets of questionnaire were administered, after which informal group discussions were held. These were preferred to FGDs because, in the small tribal communities, many individuals expressed interest in participating. These sessions, therefore, constituted general information-sharing exercises in which the research team both asked and responded to questions about HIV and other issues raised by community members.

A vignette or narrative account of a fictional woman—Pushpa—living with HIV was presented to respondents, followed by questions exploring aspects of social acceptance and exclusion. Vignettes have been found to mirror what people will do in real-life situations and to elicit more objective responses than direct questions requiring personal disclosure of stigmatizing attitudes (19,20). Questions included how stigma was perceived in society at different degrees of intimacy, from accompanying an HIV-positive person to the temple (less intimate) to inviting the person to a marriage (more intimate), to buying food prepared by someone known to be HIV-positive (most intimate). A question about whether people would do or say something to hurt someone with HIV was also included to assess perceptions of overt stigma in the communities. Questions also included information about the respondents’ own characteristics (knowledge about HIV, discussion with others and having seen someone with HIV) as well as respondents’ own responses to the stigma-related questions. The questions were formulated in the style of an EMIC (Explanatory Model Interview Catalogue) (21) that has been widely used in cultural anthropology to assess stigma toward infectious and non-communicable diseases. The methodology for the questionnaire interviews is summarized in Table 1.

**Table 1.** Summary of study methodology

Instrument	Type of data	Sampling frame	Respondents interviewed	Response rate (%)
Vignette and questions	Quantitative and qualitative	• All married females, aged 15-49 years, rural community	• 494 married females, aged 15-49 years, rural community	99
		• 50 married females, aged 15-49 years, tribal community	• 49 married females, aged 15-49 years, rural community	98
		• 100 unmarried females, aged 15-19 years, rural community	• 86 unmarried females, aged 15-19 years, rural community	86
		• 100 unmarried males, aged 15-19 years, rural community	• 100 unmarried males, aged 15-19 years, rural community	100

The vignette and questions were pretested among women and adolescents in two villages located about 50 and 70 km from the tribal and rural study communities respectively. Only minor modifications were required following the pretest.

Respondents in the rural community were selected on the basis of a village household census. All married women in the village, aged 15-49 years, were eligible for the questionnaire interviews, of whom 494 (99%) were interviewed. In total, 186 unmarried adolescents (100 boys and 86 girls), aged 15-19 years, were randomly selected from the household census. The FGD respondents were obtained by circulating announcements about forthcoming sessions, after which potential respondents were invited by the research team. The characteristics of the study subjects are presented in Table 2.

In both the study areas, the interviews were conducted in Marathi, the language of Maharashtra, by two female research assistants (RAs) under the supervision of the authors.

In the rural community, the team was complemented by one male interviewer who administered the questionnaire interviews and FGDs among males. In the tribal area, the interviewees were randomly selected from a list of married women aged 15-49 years provided by the facilitating organization. Fieldworkers from the organization accompanied the researchers to explain and translate the questions into the tribal language, where necessary. Forty-nine tribal respondents completed the interviews.

In the rural area, the FGD data were tape-recorded and analyzed by the research team following the sessions. The data from the tribal discussion groups were manually recorded to make these as unobtrusive as possible and were analyzed by the researchers after the sessions. Questionnaire data were recorded and coded manually.

Ethical clearance for the study was obtained from the University of Ottawa Research Ethics Board. Consent forms, developed in collaboration with researchers at the Agharkar Research Institute, Pune, were used for each study instrument. In the tribal communities, special efforts were made to assure that participants understood the purpose and potential utility of the study.

**Table 2.** Percentage distribution of married women (N=494), aged 15-49 years, in rural community by selected characteristics

Characteristics	Tribal women	Rural women	Adolescent girls	Adolescent boys
Age-group (years)				
15-25	49	22	100	100
>25-35	24	40	-	-
>35-49	27	38	-	-
Knowledge about HIV				
Yes	55	88	71	85
No	45	12	29	15
Discussed HIV with someone				
Yes	18	28	41	65
No	82	72	59	35
Seen HIV+ve person(s)
Yes	16	37	33	20
No	84	63	67	80

### Defining HIV-related stigma

Health-related stigma, “typically characterized by social disqualification of individuals and populations who are identified with particular health problems” (22), contributes significantly to the burden of illness (22,23,24). HIV-related stigma exemplifies several dimensions of social disqualification: it is often attributed to socially marginal behaviour, such as homosexuality, illegal sex work or substance-abuse. In India, its association with sexuality is itself stigmatizing. In understanding HIV-related stigma, it is important to examine the views of unaffected people in the community to identify barriers to disclosure and access to treatment (25) because community support has been found to positively affect HIV prevention and help-seeking (26).

## RESULTS

### HIV in the study communities

In the rural community, considerable social and economic development was observed over the longitudinal study period. Irrigation from a dam in a nearby area had led to the diversification of economic activities from subsistence to cash cropping, and men were increasingly pursuing non-agricultural occupations. Literacy among married women, aged 15-49 years, increased from 35% in 1975 to 83% in 2008, and fertility had declined to near-replacement levels. With fewer children and smaller landholdings, women had more time for domestic activities and leisure/pastimes, including watching television.

Several women in the rural community had been personally affected by HIV: some were HIV-positive, others had nursed HIV-affected relatives. Local primary health centre staff reported that an average of 10 STI cases from the village were detected each month, and one village doctor said he referred 8-10 clients a year for HIV testing. Another doctor, however, said he did not refer clients suspected to be HIV-positive for testing because they would accuse him “of speaking of dirty things.”

The tribal villages were much poorer than the rural community. Most people were landless labourers, earning below US$ 1.00 a day. There were no childcare facilities, and tribal women involved in fishing or farming often left small children in the care of older siblings. Several women said they had never visited a health facility. Staff of the facilitating organization said that HIV is a hidden epidemic in the area, partly due to increasing unemployment and an upsurge of sex work and that many young people had died of AIDS-related illnesses. Staff of the organization also observed that, despite the dissemination of HIV-related information in the area, tribal people were less concerned about HIV than other pressing problems (education, employment, housing, and sanitation).

### Nature of HIV-related stigma in rural and tribal communities

Qualitative information from FGDs in the rural area, group discussion in the tribal area, and qualitative comments made by respondents during questionnaire interviews were notable for several themes that complemented findings from the questionnaire interview data.

In the rural community, awareness of the causes of HIV was high, especially among men and younger women who generally provided factual and balanced information. This could be due to the relatively high prevalence of HIV in the area, to the fact that HIV had received considerable media attention and to an HIV awareness project in the high school. This project, however, had been prematurely terminated, apparently due to objections from conservative villagers.

Men were more knowledgeable about the causes of HIV than women. Though aware of how HIV is transmitted, older women were more fearful or misinformed (e.g. referring to transmission through coughing, food, or contact with corpses of HIV infected people) than younger women.

When asked whether they ever discussed HIV with others, FGD respondents of both sexes said they sometimes discussed it with friends or family members but not with their spouses. One man said, for example, “I would definitely not discuss it with my wife. She would be the last person I would speak to about it.” Especially, older women disapproved of discussing HIV with others.

The presence of HIV-affected people in the community was readily acknowledged in the FGDs by men and younger women while more probing among older women was required before they admitted having seen anyone with HIV. Men said that stigma toward people with HIV was widespread, and estimated that 90-100% of villagers would refuse food from a person with HIV. Female respondents revealed negative feelings toward buying food from someone with HIV. Answers included: “No, they will hate her because she has that disease,” and “No, I feel the food is very dirty.” Opinions of women were divided as to whether an HIV-infected woman might be invited to a wedding or other important event. Those who felt she would be invited said that her attendance would not pose any public danger whereas those who felt she would not be invited expressed misconceptions and fear of public disapproval. Most men agreed that she would be invited but that she would be seated and served separately from the other guests.

When asked whether an HIV-positive woman should be allowed to visit a temple, many FGD respondents, both male and female, agreed that “God does not discriminate against people who are ill,” and that, by visiting the temple, she would be comforted. Others, however, responded negatively. “People will tell her to leave the village like a dog, as with other AIDS-affected people,” one woman said. Many said that people with HIV would deliberately remove themselves from the community not to embarrass others as had occurred with several HIV-affected villagers in the past.

Community members described personal observations of overt discrimination against people with HIV in the village. Many mentioned an HIV-positive married couple who were rejected by the community and banished to the fields to die alone. Men spontaneously highlighted gender inequalities, saying that HIV-positive women were less accepted by the society. They noted that, although women cared for husbands who became ill with HIV, the opposite was rarely true, even when the wife was infected by her husband.

Despite general acknowledgement of stigma in the community, many cited a positive example of an educated woman, employed as a school principal in another village, who had contracted HIV from her husband. This community-wide acceptance seemed to stem from her education, the fact that she was viewed as an innocent victim, her appearance as a healthy, attractive woman, and the positive way she coped with her situation.

Religious pilgrimages were mentioned as occasions when community members tended to ignore customary sexual norms. However, “Sexual relations are rampant,” one man observed. “Some will appear to be very religious and will wear a sacred thread but they will still engage in sexual activities.” It was reported that an estimated 25% of the wives of poor migrant labourers sold sex on those occasions. “They have no idea about HIV or the need for prevention,” one man said. Another saw such events as opportunities for HIV education, including condom promotion.

Tribal respondents were much less knowledgeable about HIV than rural respondents. Their answers ranged from acceptance of the perceived status quo (“I would not socialize with an HIV-positive person because if people see me.…they will think I have AIDS too”) to complete denial of the existence of HIV (“There is no such disease”). Many said that HIV could be transmitted by touching, kissing, sharing utensils, using the same toilet, or even walking in the shadow of an infected person. Many said that people would be afraid of becoming infected by eating food prepared by someone with HIV and expressed strong opinions: “I will not take her food and I will also tell her to stay away from my family,” and, “She has to pay for her misdeeds.” Some felt that, by inviting someone with HIV to a wedding, others would become infected (“If she attends and others touch what she has touched, they will all contract AIDS from her”).

Tribal women were less likely to accept an HIV-positive person being admitted to a temple than rural women. Several argued that “she has sinned.” Generally, tribal respondents felt that people would discriminate against a woman with HIV. Several agreed, “People will feel that she has infected her husband and will keep her at a distance, be abusive toward her.” Another said, “People will tell her to stay away and not to touch anything. She is similar to a dead person. We may get her disease.”

Only a few respondents expressed more tolerant attitudes saying that people should show compassion to the HIV-affected persons. They also conceded that HIV-positive people should be allowed to go to the temple because they might be helped by divine intervention and feel more peaceful.

A small number of tribal women said they had discussed HIV with others but such discussion did not necessarily contribute to reduction of stigma. “We discuss who has got AIDS and who we need to avoid,” one woman said. Generally, women lacked information about HIV and other “women-related and body-related issues.” For example, many women did not know what a condom was or how to use it.

### Extent of HIV-related stigma in rural and tribal communities

#### Rural community

Overall, awareness of HIV in the rural community was high as also found in the FGDs: 88% of adult women could name at least one prevention method and the principal means of HIV transmission (Table 2). Adult women mentioned television as their main source of information. Two-thirds of the boys (65%) said they had discussed HIV with someone compared to 41% of the girls and 28% of married women. Of the adolescents who had discussed HIV with someone, boys mentioned only friends whereas girls mentioned friends (50%), family members (21%), and a combination of these, including the school project (29%). However, the male research assistant reported that most boys knew that condoms could prevent infection but few could explain how to use these.

**Table 3.** Percentage of married women and adolescent female and male respondents in rural community, giving non-stigmatizing answers to stigma-related questions

Question	Women % (N)	Girls % (N)	Boys % (N)
Would people buy food from Pushpa?			
Yes	36 (483)	64 (86)	61 (100)
Would you buy food from Pushpa?			
Yes	59 (490)	84 (86)	84 (100)
Will Pushpa be invited to the wedding?			
Yes	60 (463)	79 (86)	76 (99)
Should Pushpa be invited to the wedding?			
Yes	79 (488)	98 (86)	92 (98)
Will Chhaya accompany Pushpa to the temple?			
Yes	59 (480)	81 (85)	75 (100)
Should Chhaya accompany Pushpa to the temple?			
Yes	79 (486)	95 (86)	88 (100)
Would people say or do anything to hurt Pushpa?			
No	26 (478)	46 (86)	12 (100)

The responses of women and adolescents to the stigma-related questions are presented in Table 3. Married female respondents generally perceived more stigma in the community than did the adolescents: for example, only 36% of women said that people would buy food from Pushpa compared to 64% of girls and 61% of boys.

Most respondents said they would personally buy Pushpa's food, although married women were less positive than adolescents, with 59% saying they would buy it compared to 84% of the younger groups. Those who saw no problem in purchasing the snacks from the affected person demonstrated knowledge about HIV transmission and the importance of social support whereas those who were opposed to it often referred to community-level stigma as a justification.

The majority of married respondents (60%) felt that Pushpa would be invited to her friend's wedding. Reasons included the importance of moral support and the fact that HIV is not transmitted through social contact. Others felt that Pushpa would be invited but she would decline the invitation to avoid embarrassing her friend. Again, adolescents were more positive than married women regarding this question. With respect to respondents’ own opinions about whether Pushpa should be invited, over 75% answered positively, emphasizing that HIV is not contracted through socializing with affected people.

In answer to the question about whether Pushpa's friend would accompany her to the temple, 59% of women stated that God does not discriminate and that HIV is not a sin. Several added that Pushpa should nevertheless hide her illness. To the question of whether respondents felt that her friend should go with Pushpa, most had no objection. Respondents almost unanimously agreed that people would do or say something to hurt Pushpa. Adolescent girls perceived less stigma in this regard but even among them, the majority felt that Pushpa would be stigmatized.

### HIV-related stigma in the tribal communities

Slightly over half of the tribal respondents (55%) named one method of HIV prevention—abstinence—whereas 45% were unable to mention any method (Table 2). Twenty-three percent said they had discussed HIV with someone, a percentage similar to that of rural women (28%). This finding is surprising, given that tribal women were comparatively less informed about HIV. Only 16% said they had seen someone with HIV compared to 37% in the rural community.

With regard to the vignette (Table 4), only 4% of women said that people would buy food from Pushpa. Similarly, when asked whether they themselves would buy Pushpa's food, less than 10% answered positively. Only 16% of respondents said that Pushpa would be invited to a wedding, and only 29% felt that she should be invited. Eight percent felt that a friend would accompany Pushpa to the temple, while 25% said that she should do so. The majority of tribal respondents (63%) agreed that people would say or do something to hurt Pushpa, and they appeared to endorse such behaviour.

**Table 4.** Responses to questions regarding community stigma among married women in tribal community

Question	Yes (%)	No (%)	Total (No.)
Would people buy food from Pushpa?	4	96	47
Would you buy food from Pushpa?	8	92	47
Will Pushpa be invited to wedding?	16	84	46
Should Pushpa be invited to wedding?	29	71	44
Will Chhaya accompany Pushpa to the temple?	8	92	46
Should Chhaya accompany Pushpa to the temple?	25	75	46
Would people say or do anything to hurt Pushpa?	63	37	46

## DISCUSSION

It is instructive to compare the results of this study with the larger NFHS-3 sample for Maharashtra. HIV knowledge was higher in the rural community under study than reported for rural Maharashtra (88% compared to 72% respectively). The relatively low level reported by NFHS-3 is surprising, given the extensive awareness-raising efforts undertaken throughout India. Perhaps the NFHS-3 respondents were hesitant to admit any knowledge of HIV, just as many of our female rural respondents denied, in their individual interviews, having seen an HIV-positive person (but acknowledged it in FGDs). Among scheduled tribes, 51% of female respondents in NFHS-3 had heard of AIDS (27), similar to the percentage of women in our study who could name one prevention method (55%).

The most comparable stigma-related question in the two studies concerned buying food from an HIV-positive person. In rural Maharashtra, 48% of female respondents said they would buy it compared to 59% of our rural respondents. Among tribal women, the NFHS-3 reported similar results as for non-tribal women, a finding different from ours, in which only 8% of tribal women said they would buy Pushpa's snack. The consistency in our tribal women's responses across the stigma-related questions and the strength of stigma revealed by their qualitative remarks lead us to question the reliability of this finding in the NFHS-3 data on the tribal community.

Focusing on our results, both rural and tribal people expressed many fears, including the possibility of becoming infected themselves. The hesitation of community members to use preventive measures, the lack of open discussion about sexual matters, the denial of possible personal risks, and the existence of widespread stigma suggest that HIV infections are spreading undetected in rural areas or being detected only at advanced stages of the illness. The reported rise of sex work among poor and tribal people, accompanied with the lack of HIV-related knowledge, is of particular concern.

In the rural community, older women expressed more stigma than younger women and adolescents, perhaps because they had seen more people with HIV and how the community treated them. The high awareness about HIV among adolescents is an indication of the success of the school project. Nonetheless, the fact that it was terminated prematurely suggests the need to convince influential community members about the vital importance of such interventions for the health and well-being of youths. The reliance on friends for information about sexuality, especially by boys, is problematic because such information may be incomplete or inaccurate.

Respondents systematically attributed more stigma to others than to themselves, a finding also noted elsewhere (28,29). Perhaps they hesitated to disclose their true feelings to the interviewers but even if this was true, the sentiment that one should not discriminate against people with HIV was widely expressed in the rural community and, to some extent, by tribal respondents.

Gender-related HIV stigma was not specifically investigated but several gender issues were mentioned by respondents as influencing the extent and impact of stigma. Interestingly, males, rather than females, highlighted the greater vulnerability of women who were reported to suffer additional stigma. This confirms the observations of others (8,12,30), concerning gender as a determinant of stigma for HIV-positive people in many societies. We also found that cultural taboos discouraged the discussion of sexual issues between couples as noted by others (8,31). Though informed about HIV prevention, women felt powerless to discuss it with their partners or to protect themselves.

### Conclusions

There are many barriers to combating HIV in rural India—ranging from the inability of couples to discuss or negotiate safe sexual behaviour to engagement in sex work for economic survival in poverty-stricken areas. Fear and denial in the tribal communities and an increase in high-risk behaviour indicate that tribal communities are in an early phase of an HIV epidemic as noted by others (14). A finding common to both rural and tribal populations was an acceptance of HIV-related stigma, indicating how silence is contributing to stigma in rural areas, even by those who felt it was wrong. Thus, awareness alone did not lead to positive action. The resultant silence only contributes to furthering stigma in these areas. Education, preventive measures, and interventions to reduce community-level stigma are, therefore, of paramount importance.

A more positive finding is the widespread HIV awareness in the rural community, which could represent a first step in challenging accepted stigma. In this regard, a phased approach, beginning with proven, effective measures such as concerted STI/HIV awareness campaigns, accompanied with a steady and reliable supply of condoms to village outlets (8), could be considered. Greater exposure of these communities to HIV-positive people in social settings could also help ‘normalize’ HIV infection. The example of the widespread acceptance of the educated HIV-positive teacher augurs well for this type of intervention.

Given the lack of discussion on sexual matters within families, a key component of rural school curricula should be comprehensive sex education, with objective information about reproductive health, including HIV. Community health workers, who regularly visit rural communities, could perhaps dedicate more time for HIV-related discussions.

Our findings indicate that the media, especially television, have considerable potential to raise awareness among rural people. ‘Edutainment’, which provides factual information in an entertaining way, could be a useful strategy, especially when targeted at the entire family. In tribal areas, traditional means of entertainment, including music and street-plays, could be employed to tackle issues, such as reproductive health, negotiation skills for women, and responsible sexual behaviour.

Our methodology for assessing stigma was found to be relevant in both rural and tribal areas and across age-groups. The vignette and question guides are short, simple, and easily adapted to different cultural contexts, making them potential tools for evaluating the impact of interventions to reduce community-level stigma.

## ACKNOWLEDGEMENTS

The study was supported by the Swiss Tropical and Public Health Institute and Health Canada's HIV/AIDS Global Engagement Grants Program, 2007-2008.

We are grateful to the community leaders and the many villagers who facilitated our data collection and assisted us in many ways during the fieldwork, and to our respondents for their willingness to answer our questions. We wish to thank our research assistants Ms Sangita Mohite, Ms Chhaya Sawant, and Mr. Nitiraj Sabale for conducting the interviews. We also wish to acknowledge the assistance of Mr. Rushikesh Jangam and Mr. Avinash Jamdade for data-entry. We are grateful to Mr. Adinath Omble and *Shramik* for supporting our work in the tribal area.
